# Recurrence patterns after maximal surgical resection and postoperative radiotherapy in anaplastic gliomas according to the new 2016 WHO classification

**DOI:** 10.1038/s41598-017-19014-1

**Published:** 2018-01-15

**Authors:** Jung Ho Im, Je Beom Hong, Se Hoon Kim, Junjeong Choi, Jong Hee Chang, Jaeho Cho, Chang-Ok Suh

**Affiliations:** 1 Department of Radiation Oncology, Yonsei University College of Medicine, Seoul, Korea; 2 Department of Neurosurgery, CHA Bundang Medical Center, School of Medicine, CHA University, Bundang, Korea; 3 Department of Pathology, Yonsei University College of Medicine, Seoul, Korea; 4 College of Pharmacy, Yonsei Institute of Pharmaceutical Sciences, Yonsei University, Incheon, Korea; 5 Department of Neurosurgery, Yonsei University College of Medicine, Seoul, Korea

## Abstract

We assessed the appropriateness of current radiotherapy volume for WHO grade III gliomas. The records of 73 patients with WHO grade III gliomas who received postoperative radiotherapy between 2001 and 2013 were retrospectively reviewed. Based on the 2016 WHO classification, 25/73 (34.2%) patients had anaplastic oligodendroglioma (AO), IDH-mutant and 1p/19q-codeleted; 11/73 (15.1%) patients had anaplastic astrocytoma, IDH-mutant; and 37/73 (50.7%) patients had anaplastic astrocytoma, IDH-wildtype. The extent of resection (EOR) was total in 43 patients (58.9%). The median follow-up time was 84 months. The 5-year overall survival was 65.4%. Of 31 patients with documented recurrences, 20 (64.5%) had infield gross tumor volume (GTV) failure, six (19.4%) had clinical target volume (CTV)/marginal failure, and five (16.1%) had outfield failure/seeding. In 13 recurrences among 43 patients who underwent gross total resection (GTR), six (46.2%) had infield CTV/marginal failure. However, among 30 patients for whom GTR was not conducted, infield GTV failure was dominant (77.8%). Seventeen patients with AO, IDH-mutant and 1p/19q-codeleted who underwent GTR experienced no recurrence. In conclusion, maximal surgical resection and postoperative radiotherapy resulted in a favorable prognosis, especially in patients with GTR, IDH mutation, and 1p/19q codeletion. Patterns of failure differed by EOR.

## Introduction

The current recommendation for the World Health Organization (WHO) grade III glioma treatment is maximal safe resection followed by radiotherapy (RT). Additional chemotherapy (CTx) with procarbazine, lomustine, and vincristine (PCV) has been proven to effectively prolong the survival of patients with anaplastic oligodendroglial tumors in the EORTC Brain Tumor Group Study 26951 and RTOG 9402 trials^1,2^. In both trials, initial RT volume included T2-weighted abnormality plus a 2–2.5-cm margin (45–50.4 Gy in 25–28 fractions), and boost field was T1 abnormality (enhanced lesion and/or non-enhanced mass) plus a 1–1.5-cm margin (9–14.4 Gy in 5–8 fractions; total radiation dose was 59.4 Gy in 33 fractions). At our institution, physicians perform irradiation according to the same policy. The survival outcome of grade III gliomas differs according to the histological subtypes, anaplastic astrocytoma (AA), anaplastic oligodendroglioma (AO), and anaplastic oligodendroglioma (AOA). However, recurrence patterns according to the histological subtypes have not been well studied. Most studies on recurrence patterns were mainly conducted before 1995^3–7^, and most included high-grade gliomas together with WHO grade III and IV gliomas, with a strong majority of the patients with glioblastoma. Trials for grade III tumors alone are rare, and no study has reported recurrence patterns according to histological subtypes. Although the extent of surgical resection is a strong prognostic factor^8–11^ and may affect the recurrence pattern, recurrence patterns according to surgical extent have not yet been well defined.

Recently, molecular profiling, including 1p/19q codeletion, isocitrate dehydrogenase gene (IDH) mutations, and O6-methylguanine-DNA-methyltransferase gene (MGMT) promoter methylation, were shown to be more useful in identifying the prognostic subgroups of WHO grade III gliomas^12–16^. The International Society of Neuropathology-Haarlem guidelines propose that diagnoses of central nervous system tumors should be “layered” with histological classification, WHO grade, and molecular information in the form of an integrated diagnosis to better define the disease^17^. The revised WHO Classification of Tumors of the Central Nervous System 2016 has considered these developments and released a new diagnostic concept that combines histological and molecular parameters^18^.

We hypothesized that recurrence patterns differ by extent of resection (EOR) and histological subtypes, as well as molecular subtypes. Therefore, we analyzed recurrence patterns with respect to RT target volume and compared the differences between patient characteristics, such as EOR, and the 2016 WHO classifications.

## Methods and Materials

### Study design and patient characteristics

Seventy-three patients with WHO grade III gliomas who underwent surgical resection and postoperative RT between 2001 and 2013 and were followed-up for >6 months with magnetic resonance imaging (MRI) were retrospectively analyzed. Patients who received whole-brain RT were not included in the study. None of the patients in this study had low-grade gliomas before being diagnosed with WHO grade III gliomas. The study protocol conformed to the ethical guidelines of the 1975 Declaration of Helsinki, as revised in 1983, and was approved by the institutional review board of Severance Hospital. The patient records/information were anonymized and de-identified prior to analysis; informed consent was not obtained from each participant.

The characteristics of all 73 patients are listed in Table 1. The median age was 38 years (range, 18–67 years), and 55 patients (75.3%) were <50 years old. All patients had a Karnofsky performance status of ≥70. Pathological diagnosis was made based on the 2007 WHO classification at initial diagnosis. Twenty-four patients (32.9%) had AA, 25 (34.2%) had AO, and 24 (32.9%) had AOA.

**Table 1:** Baseline characteristics of all patients.

Characteristic (n = 73)	No. of patients (%)
Age (years)	
≤50	55 (75.3)
>50	18 (24.7)
Sex	
Male	39 (53.4)
Female	34 (46.6)
Preoperative KPS	
90–100	21 (28.8)
70–80	52 (71.2)
Main presenting symptom	
Seizure	25 (34.2)
Headache	21 (28.8)
Other	27 (37.0)
Pathological diagnosis according to the 2007 WHO classification	
AA	24 (32.9)
AO	25 (34.2)
AOA	24 (32.9)
1p/19q status	
Codeletion present	32 (43.8)
Codeletion absent	41 (56.2)
IDH1 status	
Mutated	36 (49.3)
Wild type	37 (50.7)
2016 WHO classification	
AO, IDH-mutant and 1p/19q-codeleted	25 (34.2)
AA, IDH-mutant	11 (15.1)
AA, IDH-wildtype	37 (50.7)
MGMT promoter status	
Methylated	49 (67.1)
Unmethylated	24 (32.9)
Extent of resection	
Gross total resection	43 (58.9)
Subtotal resection	18 (24.7)
Partial resection	8 (11.0)
Biopsy	4 (5.5)
Chemotherapy	
Yes	45 (61.6)
No	28 (38.4)

KPS, Karnofsky Performance Status; WHO, World Health Organization; AA, Anaplastic Astrocytoma; AO, Anaplastic Oligodendroglioma; AOA, Anaplastic Oligoastrocytoma; IDH, isocitrate dehydrogenase gene; MGMT, O^6^-methylguanine-DNA-methyltransferase gene.

### Surgery

All patients underwent preoperative MRI with gadolinium enhancement. Histological diagnosis and maximal safe tumor removal was performed by specialized neuro-oncological surgeons. Postoperative MRI was performed within 48 hours after surgery. EOR was determined on the basis of the operative findings and postoperative MRI, including postcontrast T1 and T2 fluid-attenuated inversion recovery (FLAIR) images. Gross total resection (GTR) was defined as complete resection of the enhanced and unenhanced masses and no obvious residual tumor. Subtotal resection (STR) was defined as resection of a gross tumor by ≥90%. Partial resection (PR) was defined as resection of a gross tumor by >50%. GTR was achieved in 43 patients (58.9%). STR or PR was performed in 18 (24.7%) and 8 (11.0%) patients, respectively. In four patients (5.5%), only biopsy (Bx) was performed.

### Postoperative RT and CTx

RT was started 12–53 (median 22) days after surgery. Three-dimensional conformal RT was used to treat 63 patients (86.3%), and 10 patients (13.7%) were treated with intensity-modulated RT using tomotherapy. The computed tomography scan obtained at simulation and after reconstructed with a 3-mm-slice thicknesses was fused to pre- and postoperative MRI data, including postcontrast T1 images and T2 or FLAIR images. Gross tumor volume (GTV) included the resection cavity and any gross residual tumor observed on postoperative MRI plus a 0.5–1-cm margin to compensate for the irregularity and uncertainty of the margin. The clinical target volume (CTV) was defined by GTV and hyperintense area on T2 FLAIR MRI plus a 1.5–2-cm margin accounting for potential microscopic extension. The planning target volume (PTV1) was CTV plus a 3-mm margin in all directions to account for daily setup uncertainty. A subsequent boost was given to PTV2, which was defined as GTV plus a 0.3-cm margin. The median total dose of PTV1 was 46 Gy (range, 36–51) with 1.8–2 Gy per fraction. The median total dose of PTV2 was 60 Gy (range, 50–70). Three patients (4.1%) received <59 Gy.

Adjuvant CTx was administered according to the physician’s preference. Forty-five patients (61.6%) received adjuvant CTx. Twenty-two patients received RT plus concomitant TMZ followed by adjuvant TMZ, while 13 patients received RT followed by TMZ. A median of six cycles of adjuvant TMZ was administered. Eight patients received PCV CTx after completion of RT.

### Molecular biomarkers assessment

We retrospectively examined IDH1 mutation using a Ventana Bench Mark XT autostainer (Ventana Medical System, Inc., Tucson, AZ, USA) according to the protocol. The antibody used was anti-human IDH1 R132H mouse monoclonal antibody (Clone H09L, 1:80 dilution; Dianova, Hamburg, Germany). When the cytoplasmic expression of IDH1 R132H was identified in glioma cells, we considered the case as “mutant”/“positive.”

FISH analysis of 1p/19q status was performed using the LSI 1p36/1q25 and 19q13/19p13 Dual-Color Probe Kit (Abbott Molecular Inc., Abbott Park, IL, USA). Acquired images were interpreted by two neuropathologists as the basis for EURO-CNS protocols^19^. If the numbers of “deleted” nuclei exceed 50%, the tumor was considered to show a “deletion” for the targeted chromosome. MGMT gene promoter methylation was assessed by methylation-specific polymerase chain reaction as previously described^20,21^.

Thirty-two patients (43.8%) had 1p/19q codeletion, an IDH1 mutation was detected in 36 patients (49.3%), and an MGMT promoter was methylated in 49 patients (67.1%) (Table 1). Two pathologists independently re-reviewed the pathology and molecular biomarkers and confirmed the pathological diagnosis according to the 2007 WHO classification and 2016 WHO classification. We reclassified our 73 cases according to the 2016 WHO classification: 25 patients (34.2%) with AO, IDH-mutant and 1p/19q-codeleted, 11 patients (15.1%) with AA, IDH-mutant, and 37 patients (50.7%) with AA, IDH-wildtype^18^. MGMT promoter status was significantly correlated with IDH mutation, 1p/19q codeletion, and the 2016 WHO classification (p < 0.05). More than 90% of patients with the IDH mutation or 1p/19q codeletion exhibited a methylated MGMT promoter status.

### Patient follow-up and evaluations

Patients were followed up with MRI 1 month after RT, every 3 months during the first 2 years, and every 6–12 months or when disease progression was suspected thereafter. Disease progression was defined as clinical progression based on an oncologist’s interpretation of imaging and clinical status, usually resulting in the change of treatment^22^. Pseudoprogression or radiation necrosis was differentiated with true progression by subsequent MRI, which showed resolution of contrast-enhanced lesions with edema without any change in therapy^23^.

**Figure 1:**
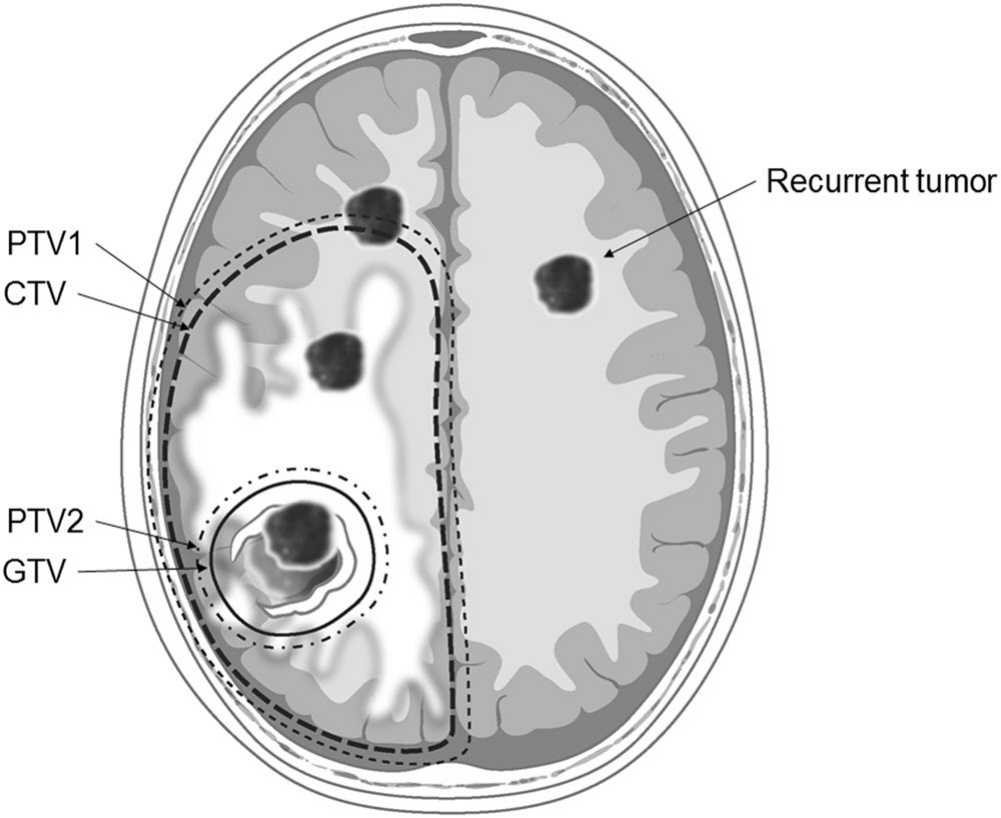
Schematic examples with the definitions of RT target volumes and patterns of recurrences. “infield GTV failure”: tumor recurrence or disease progression entirely within GTV; “infield CTV failure”: tumor recurrence within CTV; “marginal failure”: consisting of recurrent tumor crossing CTV; and “outfield failure”: in all other cases, referring to recurrences outside the RT field.

Serial MRI images were reviewed to determine first failures. All recurrence patterns were analyzed by a neuro-oncology team consisting of radiation oncologists, neurosurgeons, and neuroradiologists. Recurrences were defined as follows: “infield GTV failure” tumor recurrence or disease progression entirely within GTV; “infield CTV failure” tumor recurrence within CTV; “marginal failure” consisting of recurrent tumor crossing CTV; and “outfield failure” in all other cases referring to recurrences outside the RT field. The cerebrospinal fluid (CSF) seeding was defined as three or more non-contiguous lesions in addition to disease (stable or progression) at the primary site or leptomeningeal enhancement. Figure 1 illustrates schematic examples with the definitions of RT target volumes and patterns of recurrences.

### Statistical analysis

Progression-free survival (PFS) was calculated as the time from the date of operation to the first reported disease progression or death. Overall survival (OS) was calculated as the time from the date of operation to the date of death or last follow-up visit. Categorical variables were compared using the Chi-square test or Fisher’s exact test, as appropriate. Survival rates were calculated using Kaplan–Meier methods and compared using the log-rank test. Multivariate analysis was performed using the Cox proportional hazards model and hazard ratio with a 95% confidence interval to identify prognostic factors. Criteria for the inclusion of variables in a multivariate analysis included statistical significance on univariate analysis and clinical relevance. P values of <0.05 indicated statistical significance.

## Results

### Survival outcome and toxicity

**Figure 2:**
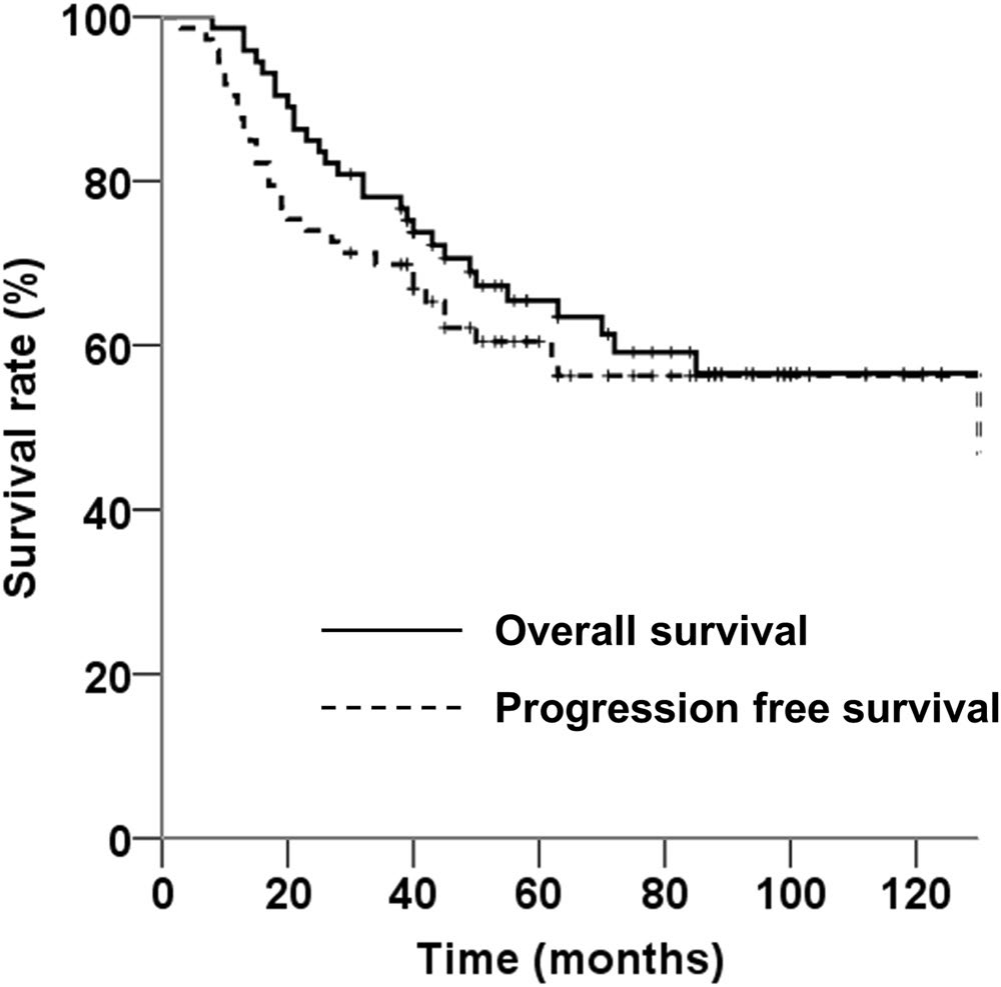
Progression-free survival and overall survival rates for all 73 patients.

The median follow-up period was 55 months (range, 8–180 months) for all patients and 84 months (range, 30–180 months) for the surviving patients. The median follow-up period of the surviving patients with AO, IDH-mutant and 1p/19q-codeleted; patients with AA, IDH-mutant; and patients with AA, IDH-wildtype was 63 months, 78 months, and 98 months, respectively. At the time of analysis, 29 patients (39.7%) had died 8–158 months after diagnosis (median, 32 months). The median PFS and OS were 130 (range, 56–204 months) and 158 months, respectively. The 5- and 10-year PFS rates were 60.4% and 56.3%, respectively, and 5- and 10-year OS rates were 65.4% and 56.6%, respectively (Fig. 2). MRI imaging abnormalities suggestive of pseudoprogression or radiation necrosis were observed in 20 patients (27.4%). No patients were diagnosed with radiation necrosis on biopsy or surgical resection. No patients died from radiation necrosis.

### Prognostic factors

**Figure 3:**
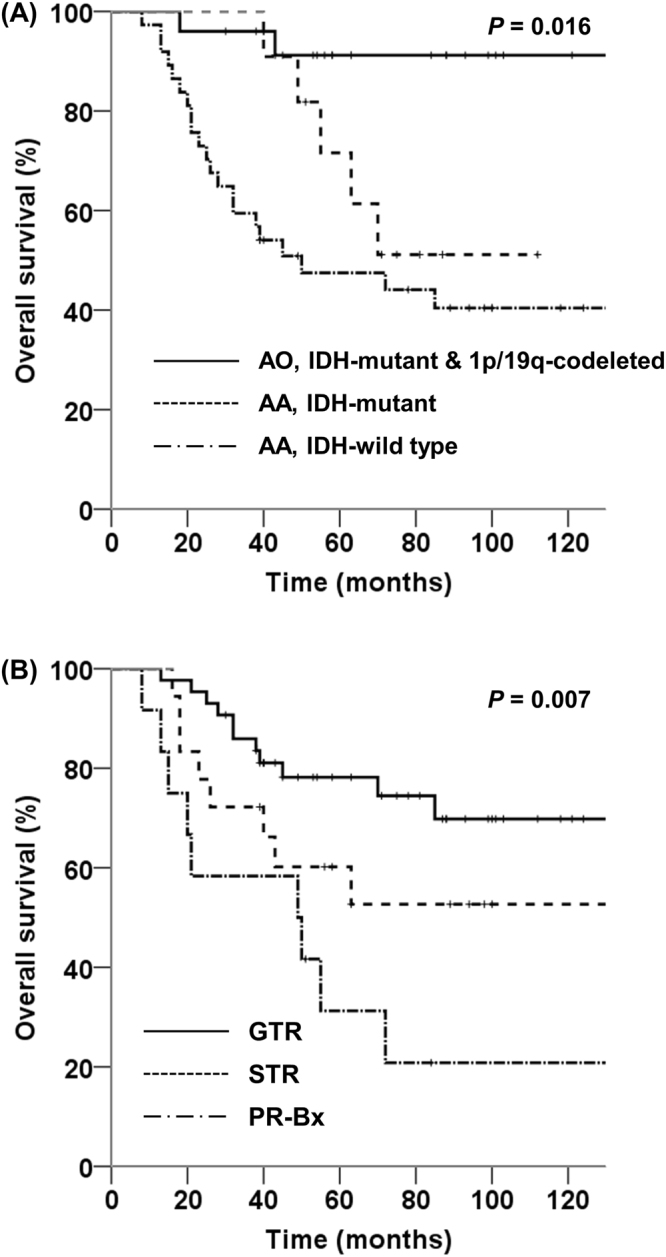
(**A**) Overall survival rate according to the 2016 WHO classification (AO, anaplastic oligodendroglioma; AA, anaplastic astrocytoma; IDH, isocitrate dehydrogenase gene). (**B**) Overall survival rate by extent of resection (GTR, gross total resection; STR, subtotal resection; PR, partial resection; Bx, biopsy).

The results of the univariate analysis of PFS and OS are summarized in Table 2. The univariate analysis showed that 1p/19q status, IDH1 status, pathological diagnosis according to the 2016 WHO classification, MGMT promoter status, and EOR were prognostic factors for PFS and OS (p < 0.05). The 5-year OS of patients with AO, IDH-mutant and 1p/19q-codeleted; patients with AA, IDH-mutant; and patients with AA, IDH-wildtype was 91.2%, 71.6%, and 47.5%, respectively (AO, IDH-mutant and 1p/19q-codeleted vs. AA, IDH-mutant, p = 0.041; AO, IDH-mutant and 1p/19q-codeleted vs. AA, IDH-wildtype, p < 0.001; AA, IDH-mutant vs. AA, IDH-wildtype, p = 0.257) (Fig. 3A). Patients with GTR had significantly better OS compared to patients with PR or Bx (p = 0.002) (Fig. 3B).

**Table 2:** Univariate analysis of prognostic factors regarding progression-free and overall survival.

Prognostic factor	No. of patients	Progression free survival			Overall survival		
		Median (m)	5/10-year (%)	p-value	Median (m)	5/10-year (%)	p-value
Age (y)				0.112			0.299
≤50	55	143	64.7/59.7		158	69.9/58.9	
>50	18	50	45.8/45.8		50	49.7/49.7	
Sex				0.245			0.416
Male	39	143	51.9/51.9		158	61.4/52.9	
Female	34	130	69.9/62.1		NR	69.4/60.3	
Preoperative KPS				0.617			0.951
90–100	21	NR	64.3/64.3		NR	64.3/64.3	
70–80	52	130	58.7/53.6		158	65.9/55.3	
1p/19q status				<0.001			<0.001
Codeletion present	32	NR	87.5/87.5		NR	90.0/90.0	
Codeletion absent	41	42	40.8/35.4		55	47.7/35.1	
IDH1 status				<0.001			0.001
Mutated	36	NR	79.4/74.5		NR	83.9/74.6	
Wild type	37	40	42.1/38.8		50	47.5/40.4	
2016 WHO classification				0.001			0.001
AO, IDH-mutant and 1p/19q-codeleted	25	NR	88.0/88.0		NR	91.2/91.2	
AA, IDH-mutant	11	NR	63.6/53.0		NR	71.6/51.1	
AA, IDH-wildtype	37	40	42.1/38.8		50	47.5/40.4	
MGMT promoter status				0.002			<0.001
Methylated	49	NR	71.0/71.0		NR	73.9/73.9	
Unmethylated	24	45	38.3/27.3		50	48.1/26.7	
Extent of resection				<0.001			0.007
Gross total resection	43	143	76.5/69.2		158	78.2/69.8	
Subtotal resection	18	NR	55.0/55.0		NR	60.2/52.7	
Partial resection-biopsy	12	28	16.7/16.7		49	41.7/20.8	
Chemotherapy				0.970			0.701
Yes	45	143	58.6/55.7		158	65.3/56.3	
No	28	130	63.0/56.0		NR	65.1/54.3	

KPS, Karnofsky Performance Status; Dx, diagnosis; WHO, World Health Organization; IDH, isocitrate dehydrogenase gene; AO, Anaplastic Oligodendroglioma; AA, Anaplastic Astrocytoma; MGMT, O^6^-methylguanine-DNA-methyltransferase gene; NR, not reached.

On multivariate analysis, GTR-STR had a significantly positive impact on PFS and OS (p < 0.05). Compared to patients with AA, IDH-wildtype, those with AO, IDH-mutant and 1p/19q-codeleted had significantly better PFS and OS (p < 0.05) (Table 3).

**Table 3:** Multivariate analysis of prognostic factors for progression-free and overall survival.

Variable	PFS		OS	
	HR (95% CI)	p-value	HR (95% CI)	p-value
2016 WHO classification				
AO, IDH-mutant and 1p/19q-codeleted	Reference		Reference	
AA, IDH-mutant	2.674 (0.618–11.580)	0.188	4.290 (0.816–22.558)	0.085
AA, IDH-wildtype	5.725 (1.688–19.412)	0.005	8.252 (1.919–35.484)	0.005
Extent of resection				
GTR-STR	Reference		Reference	
PR-Bx	3.017 (1.411–6.452)	0.004	2.405 (1.079–5.363)	0.032

WHO, World Health Organization; AO, Anaplastic Oligodendroglioma; IDH, isocitrate dehydrogenase gene; AA, Anaplastic Astrocytoma; GTR, Gross Total Resection; STR, Subtotal Resection; PR, Partial Resection; Bx, Biopsy; PFS, Progression Free Survival; OS, Overall Survival; HR, Hazard Ratio; CI, Confidence Interval.

### Recurrence rate and patterns of recurrence

Disease progression or recurrence was noted in 31 patients (42.4%) 3–143 months after diagnosis (median, 19 months). Twenty-seven of 31 failures (87.1%) occurred within 60 months after the operation. Recurrence rates and patterns of recurrences were analyzed according to the 2016 WHO classifications, as well as EOR (Table 4). Infield GTV failure was the dominant type of failure (20/31, 64.5%). Outfield failure was observed in one patient, and CSF seeding occurred in four patients.

**Table 4:** Patterns of first recurrence according to the 2016 WHO classification and extent of resection.

	Recurrence	Type of recurrence		
		Infield GTV	Infield CTV/marginal	Outfield/CSF seeding
All	31	20	6	5
2016 WHO classification				
AO, IDH-mutant and 1p/19q-codeleted (n = 25)	3 (12.0%)	2	0	1
GTR (n = 17)	0 (0.0%)	0	0	0
STR (n = 7)	2 (28.6%)	1	0	1
PR-Bx (n = 1)	1 (100.0%)	1	0	0
AA, IDH-mutant (n = 11)	5 (45.5%)	4	1	0
GTR (n = 6)	1 (16.7%)	0	1	0
STR (n = 2)	2 (100%)	2	0	0
PR-Bx (n = 3)	2 (66.7%)	2	0	0
AA, IDH-wildtype (n = 37)	23 (62.2%)	14	5	4
GTR (n = 20)	12 (60.0%)	6	5	1
STR (n = 9)	4 (44.4%)	4	0	0
PR-Bx (n = 8)	7 (87.5%)	4	0	3
Extent of resection				
GTR (n = 43)	13 (30.2%)	6	6	1
STR (n = 18)	8 (44.4%)	7	0	1
PR-Bx (n = 12)	10 (83.3%)	7	0	3

The numbers represent the number of patients with recurrences. Column 2 represents the number of patients in each subgroup with recurrence and their percentages are indicated in parentheses. GTV, Gross Tumor Volume; CTV, Clinical Target Volume; CSF, Cerebrospinal Fluid; WHO, World Health Organization; AO, Anaplastic Oligodendroglioma; IDH, isocitrate dehydrogenase gene; GTR, Gross Total Resection; STR, Subtotal Resection; PR, Partial Resection; Bx, Biopsy; AA, Anaplastic Astrocytoma.

In patients with AO, IDH-mutant and 1p/19q-codeleted, recurrences were less commonly noted (3/25, 12%) than in those with AA, IDH-mutant (5/11, 45.5%) and AA, IDH-wildtype (23/37, 62.2%). In addition, 17 patients with AO, IDH-mutant and 1p/19q-codeleted who received GTR showed no recurrence. In the 12 recurrences among 20 patients with AA, IDH-wildtype who underwent GTR, five (41.7%) had infield CTV/marginal failure.

Recurrence rate was lower in patients with GTR/STR (21/61, 34.4%) than in those with PR-Bx (10/12, 83.3%). Recurrence patterns differed by EOR. A significant difference between the extent of resection and patterns of recurrence was noted in Fisher’s exact test (p < 0.05). In the STR and PR-Bx groups, most recurrences were infield GTV failures (14/18, 77.8%) without infield CTV/marginal failures. In contrast, six (46.2%) of the recurrences were CTV/marginal failures in the GTR group.

## Discussion

Survival outcome according to the 2016 WHO classification was not previously reported because IDH analysis was not routinely performed until recently. By retrospectively examining IDH mutation, we confirmed that the prognosis of WHO grade III gliomas is largely dependent on the 2016 WHO classification. Since we administered a consistent RT volume during the study period, we could evaluate the effects of the 2016 WHO classifications as well as EOR on recurrence patterns. Although infield GTV failure was the most common pattern of failure overall, patterns of failure differed by EOR.

Recurrence rates varied according to the 2016 WHO classification. The recurrence rate was very low in patients with AO, IDH-mutant and 1p/19q-codeleted, and there were no cases of recurrence in patients with AO, IDH-mutant and 1p/19q-codeleted who underwent GTR. On the other hand, 23 of 37 patients with AA, IDH-wildtype developed recurrence, even after GTR (12/20, 60%), and the most common type of recurrence was infield GTV. These findings suggest that RT is less effective for AA, IDH-wildtype. Several studies showed that IDH mutation represents radiosensitive tumors and good prognosis^24,25^. The correlation between IDH and MGMT promoter methylation was suggested in several studies^1,26^, as observed in this study. Almost all AO patients with the IDH mutation demonstrated MGMT promoter methylation and had better prognosis. These findings may reflect the improved outcome of gliomas with IDH mutation.

Recurrence rates after GTR or STR were similar but much lower than those after PR-Bx. In patients with GTR, most recurrences (12/13) occurred in the patients with AA, IDH-wildtype, and approximately half of the recurrences was infield GTV failure and the other half was infield CTV/marginal failures (Table 4). We prescribed 46–50 Gy to CTV. Therefore, a higher radiation dose to CTV can be considered for AA, IDH-wildtype. On the contrary, only one recurrence was noted in 23 GTR patients with AO, IDH-mutant and 1p/19q-codeleted or AA, IDH-mutant. Therefore, we suggest that the current RT volume and dose prescription is appropriate for these patient groups. All patients treated with STR or PR-Bx showed progression with infield GTV or outfield/CSF seeding 3–130 months (median, 14 months) after surgery. To overcome early infield GTV recurrences, dose escalation >60 Gy to the residual tumor or a combination of radiosensitizing drugs can be considered. There is insufficient evidence to prove that hypofractionated, hyperfractionated, and accelerated RT improved local control and OS in high-grade glioma patients^27^. Dose escalation is an important scheme in radiotherapeutic strategies. However, dose-escalation would be limited due to the risk of late toxicities. The role of TMZ has been assessed in a large international trial, CATNON. Interim analysis showed that adjuvant TMZ CTx was associated with significant survival benefits in patients with newly diagnosed 1p/19q non-co-deleted anaplastic glioma^28^.

Before arguing the appropriate RT volume, the tumor should be more accurately defined on imaging. MRI T2 FLAIR hyperintensity has traditionally been accepted as the target delineation of glioma. Contrast-enhanced lesions on T1-weighted images are regarded as more malignant tumors than non-enhanced lesions, and high radiation doses are usually prescribed. Compared with glioblastomas, where GTV is defined as contrast-enhanced lesions, grade III gliomas often show mixed patterns of enhancing and non-enhancing tumor masses. Therefore, accurate delineation of tumor extent and identification of the most malignant parts are crucial for RT planning as well as surgery. Perfusion-weighted imaging is helpful to detect higher vascular tumors, which should be included in GTV^29^. Recently, amino acid PET imaging has been investigated for tumor extent and biopsy guidance; this showed that amino acid PET was superior to the standard MRI^30^. Currently, our institution uses amino acid PET for guiding tumor resection and RT planning. Metabolic imaging with MR spectroscopy to image the 2-hydroxyglutarate signal in the brain to detect oncogenic IDH1 mutations has been recently developed. It showed that the 2-hydroxyglutarate volume was larger than the FLAIR volume in approximately half of the IDH-mutant glioma patients^31^. These improved techniques may have implications for better RT planning and treatment outcomes.

The current study has important limitations that should be considered when interpreting our results. When a case was negative for IDH1 immunostaining results, we did not perform IDH1 and IDH2 sequencing as recommended by Louis *et al*.^18^. Although the incidence of IDH1 mutation, except R132H and IDH2, is very rare^32^, it may be an important limitation. Although we performed surgery and postoperative RT using consistent methods, CTx was not controlled, which may be a confounding factor. The small number of patients is another critical limitation of our study. We observed different failure rates and different recurrence patterns for the histological subtypes according to the 2016 WHO classification; however, the differences were not statistically significant, probably due to the small number of patients. Despite these limitations, compared to previous institutional studies, this is the largest single-institutional cohort study that analyzed the failure patterns of grade III glioma patients. In addition, to the best of our knowledge, this is the first study that analyzed the treatment outcomes using the new 2016 WHO classification.

In conclusion, maximal surgical resection and postoperative RT, including peritumoral edema, resulted in a favorable prognosis, especially in patients with GTR, IDH mutation, and 1p/19q codeletion. We observed that patterns of failure differed according to EOR. Infield GTV failures and infield CTV/marginal failures were not uncommon in patients with AA, IDH-wildtype even after GTR. Further studies investigating the optimal RT volume and dose for AA, IDH-wildtype are needed.
